# Adsorption performance and mechanism of Li^+^ from brines using lithium/aluminum layered double hydroxides-SiO_2_ bauxite composite adsorbents

**DOI:** 10.3389/fchem.2023.1265290

**Published:** 2023-10-25

**Authors:** Cheng Qian, Mianping Zheng, Yongsheng Zhang, Enyuan Xing, Baoling Gui

**Affiliations:** ^1^ Institute of Mineral Resources, Chinese Academy of Geological Sciences, Beijing, China; ^2^ Key Laboratory of Saline Lake Resources and Environment, Ministry of Land and Resources, Beijing, China

**Keywords:** LDH-Si-BX, brines, Li + adsorption behavior, mechanism, regeneration

## Abstract

A combined method of solid-phase alkali activation and surface precipitation was used to prepare the lithium/aluminum layered double hydroxides-SiO_2_ loaded bauxite (LDH-Si-BX) and applied to adsorb Li^+^ in brines. In the study, various characterization techniques such as SEM, XRD, BET, Zeta potential, and x-ray photoelectron spectroscopy (XPS) were applied to characterize and analyze the adsorbents. The adsorption-desorption performance of LDH-Si-BX for Li^+^ in brines was systematically investigated, including adsorption temperature, adsorption time, Li^+^ concentration, and regeneration properties. The results indicated that the adsorption kinetics were better fitted by the pseudo-second-order model, whereas the Langmuir model could match the adsorption isotherm data and the maximum Li^+^ capacity of 1.70 mg/g at 298K. In addition, in the presence of coexisting ions (Na^+^, K^+^, Ca^2+^, and Mg^2+^), LDH-Si-BX showed good selective adsorption of Li^+^, and the pH studies demonstrated that the adsorbents had better Li^+^ adsorption capacity in neutral environments. In the adsorption process of real brines, LDH-Si-BX had a relatively stable adsorption capacity, and after 10 cycles of adsorption and regeneration, the adsorption capacity decreased by 16.8%. It could be seen that the LDH-Si-BX adsorbents prepared in this report have the potential for Li^+^ adsorption in brines.

## 1 Introduction

As an important strategic resource, lithium and its compounds are widely used in ceramics, alloys, batteries, glass, nuclear power, and optoelectronic technology (Hu et al., 2019; Liu et al., 2019; Xiong et al., 2022). In recent years, with the continuous depletion of fossil energy, people have become concerned about energy, the environment, and sustainable development (He et al., 2021; Liu et al., 2023). Accordingly, the new energy industry, especially lithium-ion new energy has attracted widespread attention. With the booming development of the new energy industry, the consumption and demand of lithium in the battery industry have been increasing year by year, and the supply-demand conflict of lithium resources is also increasingly prominent in the world (Pramanik et al., 2020; Sun et al., 2020; Liu et al., 2021). Studies have shown that lithium resources in nature mainly include lithium-bearing solid ores and lithium-bearing brines, and in China, lithium resources mainly exist in brines, therefore, the recovery of lithium from brines possesses great economic value (Jiang et al., 2021; Liu et al., 2020). Currently, researchers have used various methods and processes to extract lithium from brines, such as precipitation (Zhang et al., 2019; Zhang et al., 2022a; Zhang et al., 2022b), electrochemistry (Mu et al., 2021), extraction (Ji et al., 2016; Xiang et al., 2017; Zhou et al., 2020), membrane separation (Wang et al., 2021; He et al., 2022), and adsorption (Marthi et al., 2021; Qian et al., 2021; Luo et al., 2021). Among the extraction methods, the adsorption method is gradually becoming a promising route for lithium extraction from brines because of its low cost, high efficiency, and ease of operation (Zhang et al., 2022a).

Among the multitude of lithium-ion adsorbents that have been developed, lithium manganese oxide (Ding et al., 2021; Ryu et al., 2022), lithium titanate oxide (Gu et al., 2018; Zhao et al., 2021), and lithium aluminum chloride layered double hydroxide (Liu et al., 2018; Zhong et al., 2021) have been extensively studied. Although lithium titanate oxide and lithium manganese oxide have higher adsorption capacity, the loss of dissolution during desorption by acid washing is serious, which greatly limits their applications (Orooji et al., 2022). Layered lithium aluminum double hydroxides (Li/Al-LDHs)have lower Li^+^ adsorption capacity than lithium titanate oxide and lithium manganese oxide, but the elution of lithium ions can be accomplished using neutral deionized water and the adsorbent does not dissolve, reflecting the great potential of Li/Al-LDHs for industrial lithium extraction (Zhong et al., 2020).

Currently, numerous research teams have prepared Li/Al-LDHs by solid-phase, co-precipitation, and hydrothermal synthesis methods, however, the adsorbents prepared by such direct synthesis using chemical reagents are mostly nanoscale powders, which are relatively costly and difficult to separate and recover in practical applications, but granulation will not only further increase the manufacturing cost, but also significantly decrease the adsorption performance of the adsorbents.

Because of the above problems, the authors used natural minerals as raw materials to prepare lithium ion adsorbent. Bauxite, a natural mineral and the natural source of aluminum, has the characteristics of being rich in aluminum, hard, low cost, and easy to obtain (Alhassan et al., 2020; Sun et al., 2022), therefore, we prepared a Li/Al-LDHs bauxite composite lithium ion adsorbent by using bauxite as the raw material and studied its adsorption kinetics, adsorption isotherm, the effects of pH and interfering ions on Li^+^.The adsorption capacity and recycling performance of Li/Al-LDHs bauxite composite for lithium ions in real brine were analyzed. The adsorption materials were characterized and analyzed by various means, and the adsorption mechanism was analyzed. We suggest that this work can provide a reference for the application of bauxite in the extraction of lithium from brine.

## 2 Materials and methods

### 2.1 Materials and reagents

The bauxite used in this study was obtained from an alumina plant in Shanxi Province, China. The chemical composition and loss on ignition (LOI) of the diasporic bauxite are listed in Supplementary Table S1. Before use, it was washed, dried, and smashed to pass through 100 mesh sieves. Deionized (DI) water was used throughout all the experiments. The chemicals used in the experiments were analytical grade and obtained from Sinopharm Chemicals Reagent Co., Ltd. (Shanghai, China).

### 2.2 Preparation of the absorbent

**FIGURE 1:**
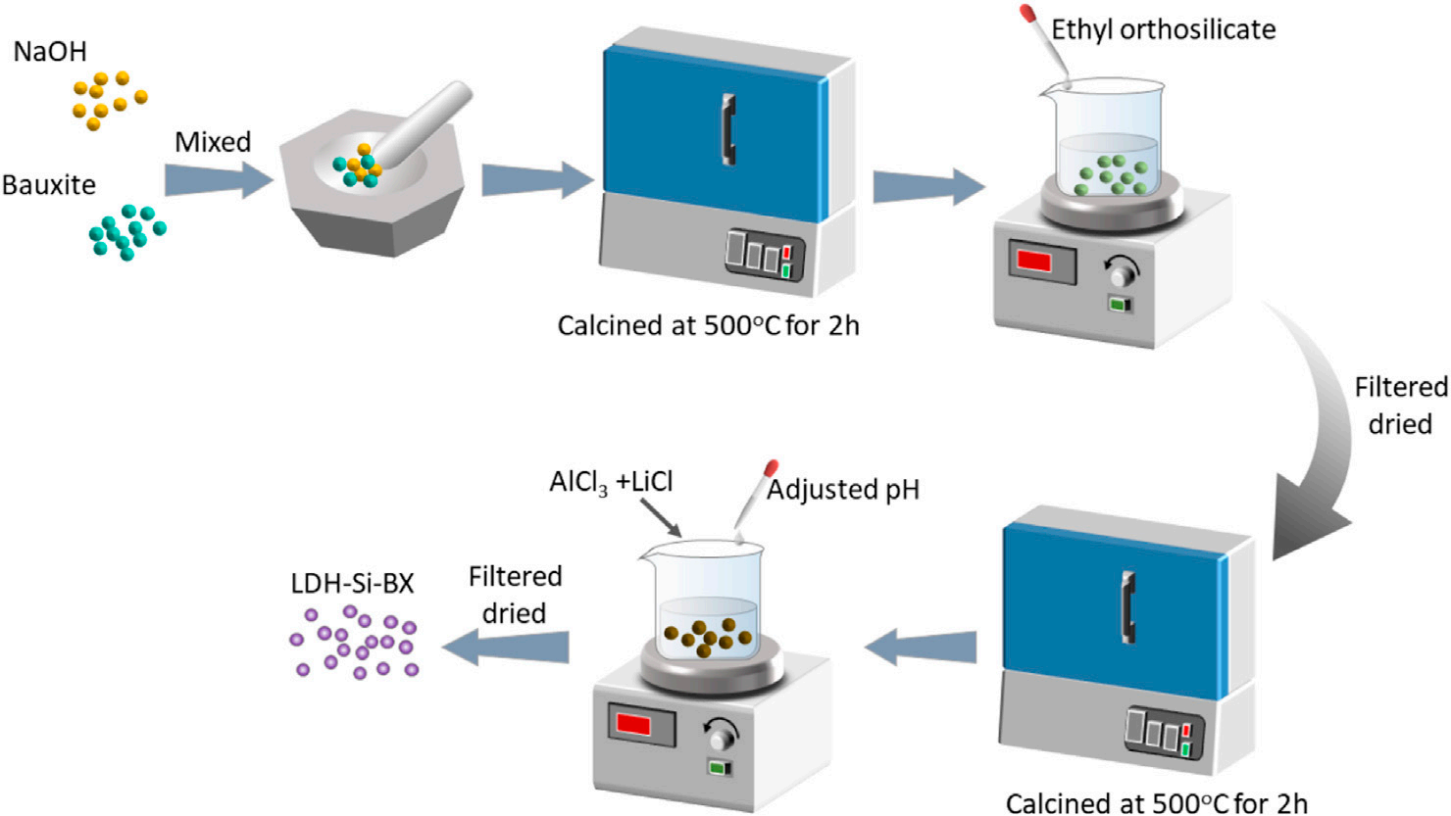
Schematic diagram of synthesized LDH-Si-BX.

Lithium/aluminum layered double hydroxides-SiO_2_ bauxite (LDH-Si-BX) was prepared as the following procedure illustrated in Figure 1.

1) 50 g natural bauxite was crushed so that we could collect 80–120 um particles. The particles were mixed with 10 g NaOH, placed in a nickel crucible at 500°C with a heating rate of 5 ^°^C/min for 2h, and then cooled to room temperature.
2) The activated bauxite was mixed with 10 mL ethyl orthosilicate for 1 h. After atmospheric filtration, the solid phase was dried at 60°C to a constant weight and placed in a nickel crucible. Heat treatment was performed under the same conditions as in step 1) and recorded as Si-BX for reserve use.
3) Dissolved 4.2 g LiCl and 13.3 g AlCl_3_ in 250 mL deionized water was mixed and stirred with Si-BX for 30 min, pH was adjusted to neutral by dropping 1 mol/L NaOH and 1 mol/L HCl, and the mixture was stirred again for 3 h. After filtration and washing, the solid phase was dried at 60°C to constant weight and denoted as LDH-Si-BX.

### 2.3 Adsorption and regeneration in a batch system

The batch test for Li^+^ adsorption was performed in 150 mL conical flasks shaken in a temperature-controlled oscillator. All the Li^+^ solution was collected by filtering through a syringe equipped with a 0.45 μm PES filter head. In the case of a lack of special instructions, the adsorption conditions were controlled at an absorbent dosage of 0.1 g, Li^+^ solution volume of 25.00 mL, room temperature, pH = 7.0, and oscillation rate of 150 rpm. In addition, the pH was adjusted by using 1 mol/L HCl or 1 mol/L NaOH.

To evaluate the kinetics of Li^+^ adsorption onto the absorbents, the absorbents were added to the tapered bottles equipped with 20 mg/L Li^+^ solution. The water samples were collected at 20, 40, 60, 90, 120, 150, 180, and 240 min, and the Li^+^ concentration was analyzed.

The Li^+^ adsorption isotherm was investigated by adding the adsorbents to different concentrations (5, 10, 25, 50, 75, and 100 mg/L) of Li^+^ solution. After adsorption for 24 h, the Li^+^ concentration was tested and calculated.

To further investigate the lithium-ion adsorption performance and selectivity under coexisting ion conditions, the adsorption process was conducted in the solutions of Na^+^, K^+^, Ca^2+,^ and Mg^2+^ with an initial concentration of 250 mg/L, which is five times that of Li^+^. The absorbents were then additionally oscillated into the above solution and adsorbed to achieve equilibrium, and the Li^+^ content of the remaining solution was detected.

To examine Li^+^ adsorption properties under different pH levels, the adsorbents were mixed with 20 mg/L Li^+^-containing solutions of different pH values ranging from 3.0 to 11.0. After oscillating for 24 h, Li^+^ content and pH value were determined for the remaining solution.

To further study the adsorption-desorption performance of Li^+^ in real brines, 10.0 g LDH-Si-BX was added to 250 mL brines and adsorbed for 6 h to detect the content of lithium ions in brine and calculate the adsorption capacity. During the desorption process, DI water was selected as the desorption solution. For desorption, the adsorbent was loaded into a single glass column with a porous ceramic plug at the bottom and continuously rinsed with DI water until the concentration of lithium in the effluent solution was stabilized.

### 2.4 Characterization and analysis method

Surface morphologies of the samples were obtained by field emission scanning electron microscope (FESEM, SUPRA 40, ZEISS, Germany). The physical structure including specific surface areas (S_BET_) and pore structure was assessed by using a BET surface area analyzer (TriStar II Plus 2.02, Micro, United States). Zeta Potential Analyzer (Zeta, ZS-90, Malvern, UK) was used to measure the surface charge of absorbents. The Li^+^ concentration was tested by an inductively coupled plasma optical emission spectrometer (ICP-OES, Optima 8000, PerkinElmer, United States). The chemical environment and shift of the absorbents were determined by X-ray photoelectron spectroscopy (XPS, Thermo Fisher Scientific Instrument Co., United States).

## 3 Results and discussion

### 3.1 Characterization

**FIGURE 2:**
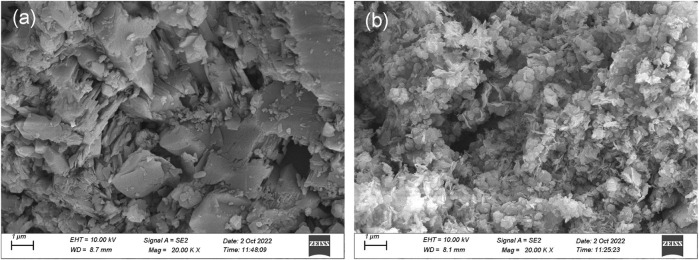
SEM images of BX **(A)** and LDH-Si-BX **(B)**.

The investigations of the surface morphology of bauxite (BX) and LDH-Si-BX after Li^+^ adsorption were performed by scanning electron microscope (SEM) and transmission electron microscope (TEM). It was found that the BX (Figure 2A) exhibited a typical flat lamellar surface and presented a blocky structure accumulated by a number of lamellas. However, the surface morphology of LDH-Si-BX (Figure 2B) dramatically differed from BX; the main differences were manifested in that the lamellar structure at the edges of the particles was covered and presented some flattened particles and scattered nanosheets, due to the loading of SiO_2_ and LDH.

**FIGURE 3:**
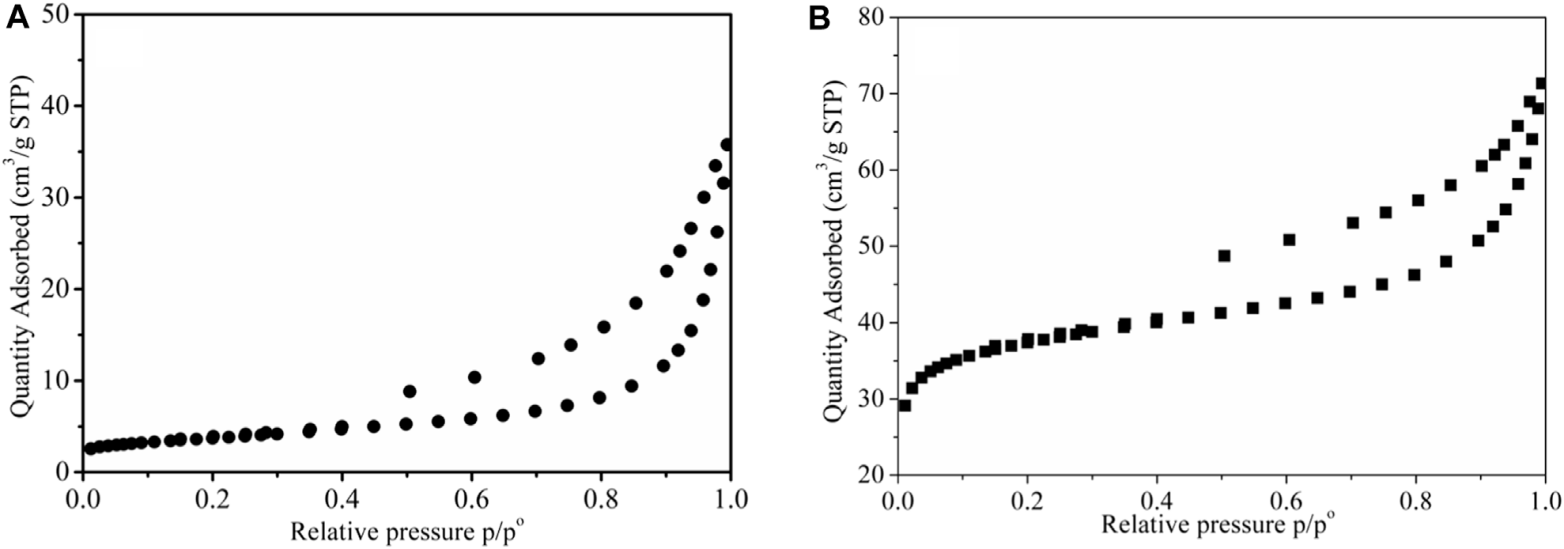
Nitrogen adsorption-desorption isotherms of BX **(A)** and LDH-Si-BX **(B)**.

The pore structures of the adsorbents before and after the modification were identified by BET surface area analysis. Figure 3 shows the N_2_ adsorption-desorption isotherm profiles for BX(a) and LDH-Si-BX(b), and the inset shows the pore size distribution, specific surface area, average pore size, and total pore capacity as shown in Table 1. As can be seen from Figure 3, for both BX and LDH-Si-BX, the adsorption-desorption isotherms belong to type IV adsorption isotherms in the IUPAC classification, and hysteresis occurs when the relative pressure *P/P*_*0*_ > 0.4 for both, indicating the existence of certain mesopores in both BX and LDH-Si-BX. As shown in Table 1, the specific surface area, average pore size, and total pore volume were 13.2 m^2^/g, 16.6 nm, and 0.05 cm^3^/g for BX and 152.6 m^2^/g, 3.1 nm, and 0.11 cm^3^/g for LDH-Si-BX, respectively. After modification, the average pore size of the adsorbent decreased, while the specific surface area and total pore volume increased significantly. The reason for this change might be the introduction of new substances in the structure of BX after loading SiO_2_ and LDH, which caused the structure to become rougher.

**TABLE 1:** The physical parameters of the prepared samples.

Sample	S_BET_ (m^2^/g)	Average pore size (nm)	Total pore volumes (cm^3^/g)
BX	13.2	16.6	0.05
LDH-Si-BX	152.6	3.1	0.11

**FIGURE 4:**
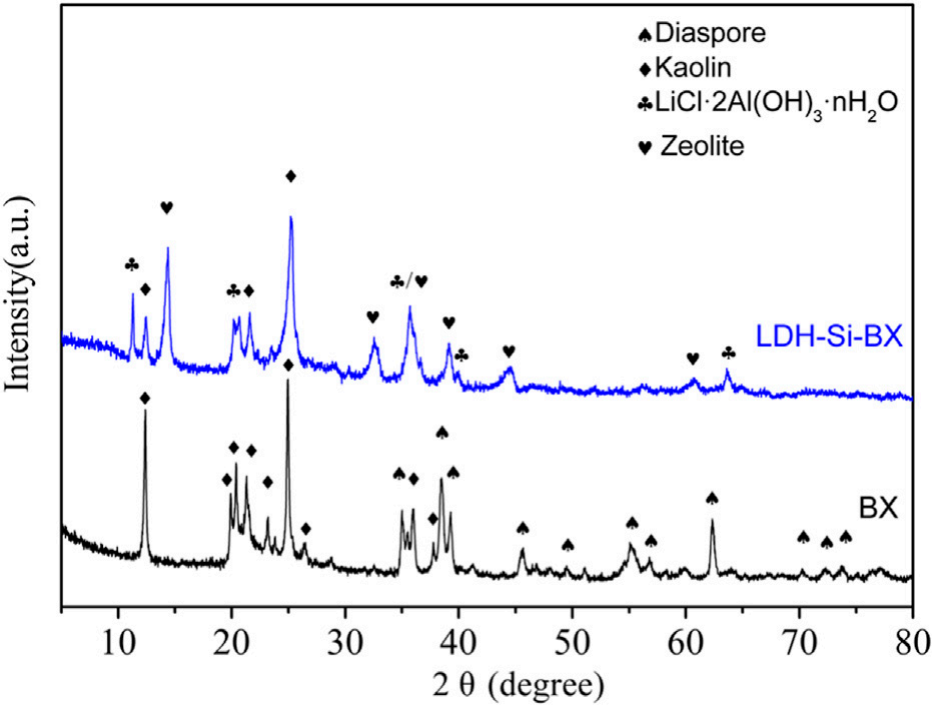
The XRD patterns for BX and LDH-Si-BX.

The XRD results of the BX and LDH-Si-BX are shown in Figure 4. BX was mainly composed of diaspore (PDF#05–0355) and kaolin (PDF#29–1,488), and the diffraction peaks of the images were narrow and sharp, indicating that BX had high order and good crystallinity. It was noteworthy that the XRD spectra of LDH-Si-BX obtained after the activation modification showed the disappearance of the hydrargyrite diffraction peaks, the decrease of kaolinite intensity, and the appearance of diffraction peaks of LiCl·2Al(OH)_3_·nH_2_O(PDF#31–0700) and zeolite (PDF#16–0612), which was attributed to the destruction of the crystalline state of kaolin and the conversion of diaspore into new substances under alkali thermal calcination conditions. In the combined effect of acid impregnation, AlCl_3_, LiCl, and NaOH, the BX structure was rearranged and partially transformed into a zeolite structure and loaded with LiCl·2Al(OH)_3_·nH_2_O. It was demonstrated that that LiCl·2Al(OH)_3_·nH_2_O had the ability to selectively adsorb Li^+^, therefore, the appearance of LiCl·2Al(OH)_3_·nH_2_O implied that LDH -Si-BX had the potential to adsorb Li^+^.

### 3.2 Adsorption kinetics

**FIGURE 5:**
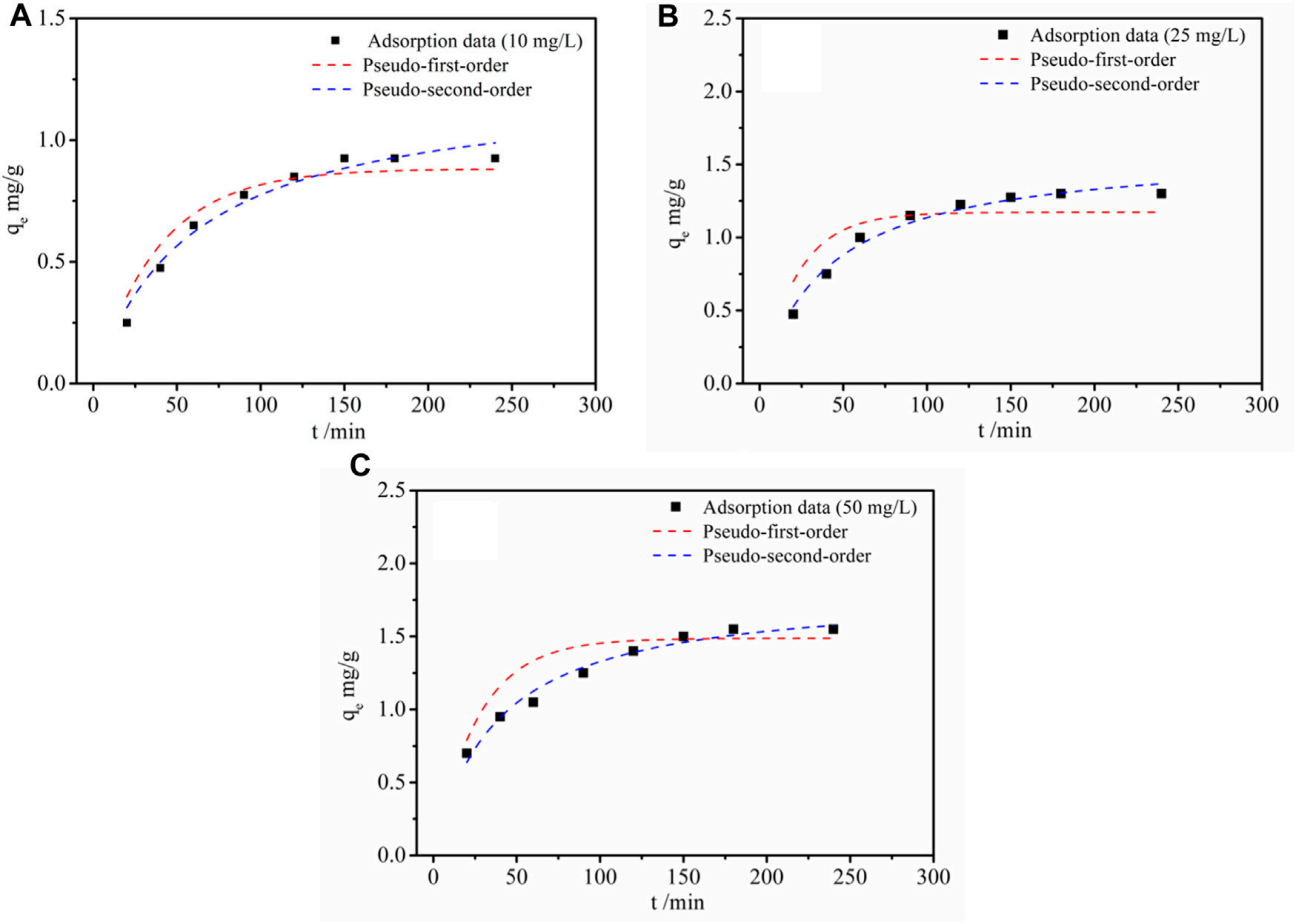
Adsorption kinetic of Li^+^ by LDH-Si-BX: 10 mg/L **(A)**, 25 mg/L **(B)** and 50 mg/L **(C)**.

To study the effect of adsorption time, a kinetic investigation was carried out. As illustrated in Figure 5, the Li^+^ adsorption capacity of LDH-Si-BX increased dramatically and the adsorption rate was fast during the first 125 min. Subsequently, the lithium adsorption capacity of LDH-Si-BX reached more than 80% of its equilibrium adsorption capacity. However, in the subsequent adsorption process, the adsorption rate began to slow down and reached equilibrium. The rapid adsorption phase might be due to the instant contact between the plentiful adsorption sites and adsorbate. When the active sites were gradually occupied, the rate-limiting step of the adsorption process was mainly the diffusion of Li^+^ into the absorbents, which resulted in the plateau. Besides, it is noteworthy that the higher initial concentration exhibited a higher uptake of Li^+^ capacity (1.54 mg/g at 50 mg/L) than that at a lower concentration condition (1.32 mg/g at 25 mg/L and 0.96 mg/g at 10 mg/L), respectively.

To make a further assessment of the adsorption process, the adsorption data derived from the experiment were modeled by using the pseudo-first-order and pseudo-second-order models (Chen et al., 2020; Kamran and Park, 2022). The mathematical expressions are individually shown in Equation 1 and Equation (2):

$$q_{t}=q_{e}\left(1-\exp\left(-k_{1}t\right)\right)$$ (1)

$$q_{t}=\frac{k_{2}q_{e}^{2}t}{1+k_{2}q_{e}t}$$ (2)

where *t* is the adsorption time (min), *q*_*t*_ is the Li^+^ adsorption capacity at the time of *t* (mg/g), *q*_*e*_ is the equalized adsorption capacity (mg/g), and *k*_*1*_ (1/min) and *k*_*2*_ (g/(mgmin)) are considered to be the adsorption rate constant of the pseudo-first-order model and pseudo-second-order model, respectively.

From Figure 5 and Table 2, it was observed that the pseudo-second-order kinetic fitting factor values of *R* ^2^ were 0.966, 0.972, and 0.974, and higher than those calculated from the pseudo-first-order kinetic model (0.892, 0.884, and 0.943). Furthermore, the calculated saturated adsorption capacity of 10 mg/L, 25 mg/L, and 50 mg/L from the pseudo-second-order kinetic model (1.16, 1.52, and 1.76 mg/g) were closer to the experimental ones of 0.96 mg/g, 1.32 mg/g, and 1.54 mg/g. Therefore, chemisorption might be presumed to be the rate-limiting step (Cheng et al., 2021).

**TABLE 2:** Kinetic parameters for Li^+^ adsorption on the absorbents.

Concentration (mg/L)	Pseudo-first-order model		Pseudo-second-order model			Experimental q_exp_ (mg·g^-1^)
	k_1_ (min^-1^)	*R* ^2^	k_2_ (g/(mg·min))	q_e_ (mg/g)	*R* ^2^	
10	0.026	0.892	0.013	1.16	0.966	0.96
25	0.035	0.884	0.015	1.52	0.972	1.32
50	0.037	0.922	0.014	1.76	0.974	1.54

### 3.3 Adsorption isotherms and thermodynamics

To further describe the Li^+^ adsorption capacity of LDH-Si-BX under various concentrations, the data gained from adsorption experimental procedures were matched by the Langmuir and Freundlich models (Wang et al., 2016; Zhang et al., 2017). The non-linear forms are illustrated by the Equations (3) and (4), respectively.

$$q_{e}=\frac{q_{\max}K_{L}C_{e}}{1+K_{L}C_{e}}$$ (3)

$$q_{e}=K_{F}{C_{e}}^{1/n}$$ (4)

where *C*_*e*_ and *q*_*e*_ are the Li^+^ concentration of the solution (mg/L) and the corresponding adsorption capacity when achieving equilibrium (mg/g), respectively, *q*_max_ (mg/g) is the saturated adsorption capacity calculated by the Langmuir adsorption model, *K*_*L*_ (L/mg) is the constant of the Langmuir isotherm model, *K*_*F*_ (mg/g) is the Freundlich constant, and *n* is the Freundlich coefficient.

To determine whether the adsorption process is facilitated or not, the infinitesimal isolation factor *R*_*L*_ was expressed as follows (Li et al., 2014).

$$R_{L}=\frac{1}{1+K_{L}C_{0}}$$ (5)

where *C*_*0*_ is the highest initial Li^+^ concentration (mg/L) and *K*_*L*_ is the constant of the Langmuir isotherm model (L/mg).

**FIGURE 6:**
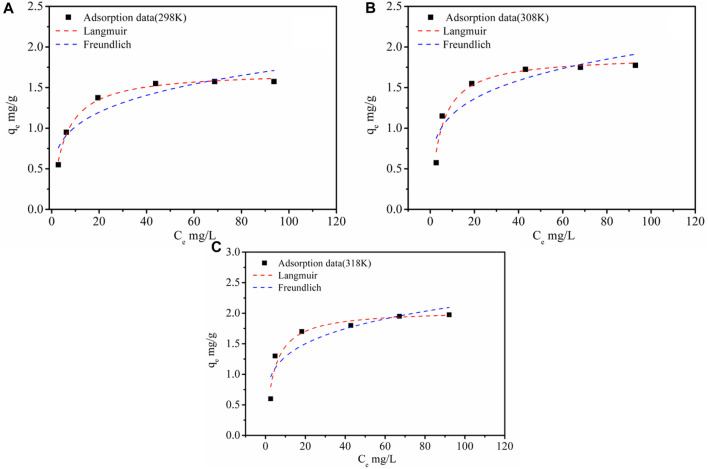
Adsorption isotherms of Li^+^ by LDH-Si-BX: 298K**(A)**, 308K**(B)** and 318K**(C)**.

The fitting results and the Li^+^ adsorption at different concentrations are given in Table 3 and Figure 6. It was shown that with an increase of initial Li^+^ concentration, the equilibrium concentration gradually increased, while the equilibrium adsorption capacity of Li^+^ presented a variation trend of fast increase, then a slight increase, and finally tended to remain constant. It might be because the limited active sites of the adsorbent were occupied by Li^+^. As can be seen from Table 3, the Langmuir model had a higher fitting factor (*R* ^2^ = 0.991 at 298K, *R* ^2^ = 0.962 at 308K, and *R* ^2^ = 0.926 at 318K), which gave a better description of the adsorption process than that of the Freundlich model. This demonstrated a process similar to monolayer adsorption that occurred during the adsorption of Li^+^ by LDH-Si-BX (Huang et al., 2015). In addition, the infinitesimal isolation factor *R*_*L*_ was in the range of 0–1 for both LDH-Si-BX, indicating that these adsorption processes were favorable. Moreover, the calculated saturated Li^+^ adsorption capacities from the Langmuir model by LDH-Si-BX were 1.70 mg/g, 1.88 mg/g, and 2.06 mg/g at 298 K, 308 K, and 318 K, respectively. As can be seen, LDH-Si-BX had a certain adsorption capacity for Li^+^.

**TABLE 3:** Parameters obtained from the Langmuir and Freundlich models for Li^+^ adsorption on the absorbents.

Temperature (K)	Langmuir model				Freundlich model		
	K_L_ (L/mg)	q_max_ (mg/g)	R_L_	*R* ^2^	K_F_ (mg/g)	n	*R* ^2^
298	0.192	1.70	0.094	0.991	0.598	4.316	0.844
308	0.224	1.88	0.082	0.962	0.704	4.536	0.798
318	0.242	2.06	0.076	0.926	0.780	4.626	0.780

**FIGURE 7:**
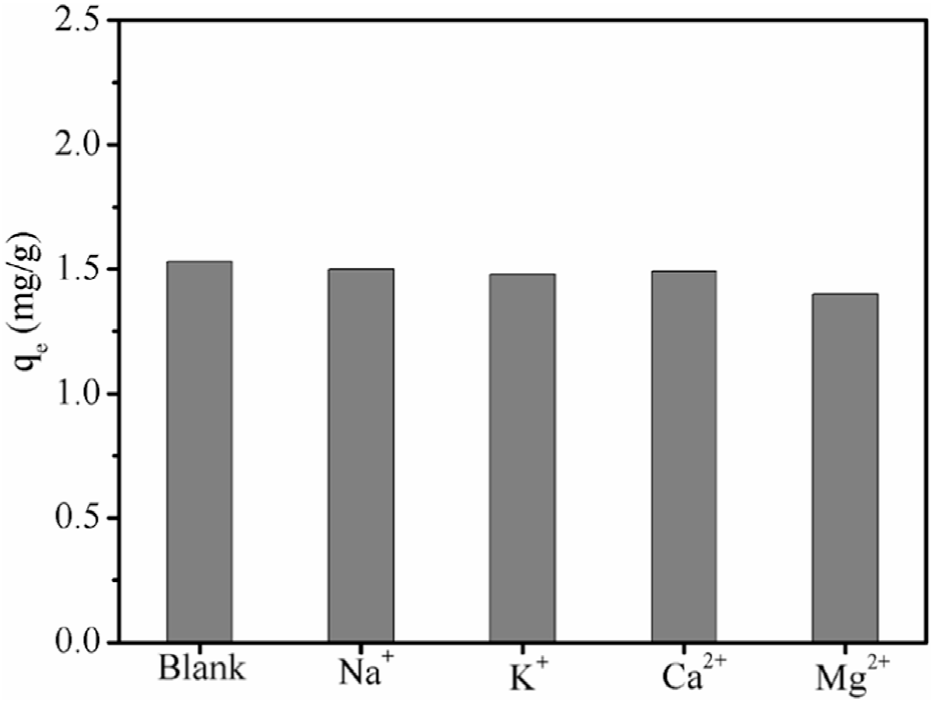
Effect of coexisting anions on the Li^+^ adsorption capacity of LDH-Si-BX.

In order to identify the feasibility and spontaneity of the Li^+^ adsorption by LDH-Si-BX, different thermodynamic parameters including enthalpy (Δ*H* ^*o*^), entropy (Δ*S* ^*o*^), and Gibbs free energy (Δ*G* ^*o*^) were obtained by linear fit with ln*K* as a function of 1/T (Figure 7B). The equations are expressed as follows (Chen et al., 2020):

$$K=\frac{1000M_{Li}\left[Li^{+}\right]}{Y}K_{L}$$ (6)

$$\Delta G^{0}=-RT\ln K$$ (7)

$$\ln K=-\frac{\Delta H^{0}}{RT}+\frac{\Delta S^{0}}{R}$$ (8)

where *K* is the dimensionless thermodynamic equilibrium constant, *M*_*Li*_ is the molecular weight of Li (6.941 g/mol), [Li^+^] is the standard concentration of adsorbate and is equal to 1 mol/L, and Y is the activity coefficient (dimensionless). The Li^+^ concentration of experimental brine in this paper is less than 100 mg/L, thus it can be considered that the adsorbate Li^+^ is very diluted and the value of the activity coefficient is assumed to be unitary. R and *T* denote the distribution, ideal gas constant (8.314 J mol^-1^·K^−1^), and temperature (K), respectively. 

Thermodynamic parameters are summarized in Supplementary Table S2. It is observed from the table that the values of ΔG are negative at 298 K, 308 K, and 318 k: 17.826, −18.208, and −18.399 kJ/mol, respectively, In addition, the positive values of enthalpy change Δ*H* ^*o*^ (9.602 kJ/mol) and entropy change Δ*S* ^*o*^ (92.102 J/(molK)) indicated that the adsorption of Li^+^ by LDH-Si-BX was an endothermic and increasing disorder degree process (Chen et al., 2020).

### 3.4 Effect of coexisting ions

Various cations are always present in the actual brines, which may affect the ability of the adsorbent to remove Li+. In the present study, we independently investigated the effects of Na^+^, Mg^2+^, K^+,^ and Ca^2+^on Li + adsorption by LDH-Si-BX. As shown in Figure 7, the presence of Na^+^, K^+^, Ca^2+,^ and Mg^2+^ showed a certain degree of decrease in the adsorption of Li ions, i.e., when the concentration of coexisting ions was five times that of Li^+^, the adsorption capacity of Li^+^, Na^+^, K^+^, Ca^2+^, and Mg^2+^, was 1.53 mg/g, 1.50 mg/g, 1.48 mg/g, 1.49 mg/g, and 1.41 mg/g, respectively, which showed that Mg^2+^ had a more obvious effect on Li^+^ in the adsorption process.

In the co-existing cation effect experiments, the adsorption process was conducted in the solutions with different initial concentrations of *Li* ^*+*^ */Me* (*Me* = Na^+^, K^+^, Ca^2+^, and Mg^2+^). The distribution coefficient (*K*_*d*_) and separation factor (*α*_*Li*_ */*_*Me*_) were determined by the following equations:

$$K_{d}=Q_{e}/C_{e}$$ (9)

$$\alpha_{Me}^{Li}=K_{d,Li}/\;K_{d,Me}$$ (10)

where *Q*_*e*_ (mg/g) is the ion equilibrium adsorption capacity of the adsorbent and *C*_*e*_ (mg/L) is the residual cation concentration in the solution.

The relevant parameters are listed in Table 4. The results indicate that the ion-selective sequence of LDH-Si-BX followed the order of Na^+^ > K^+^> Ca^2+^> Mg^2+^ with the *α*_*Li*_ */*_*Me*_ in a range of 27.98–54.34. It can be seen that LDH-Si-BX had selective adsorption of Li^+^, therefore, the use of LDH-Si-BX for selective absorption of Li^+^ in actual brines possesses some potential applicability.

**TABLE 4:** Adsorption selectivity of Li/Al-LDHs between Li^+^ and other cations.

Ions	C_0_ (mg/L)	C_e_ (mg/L)	*Q*_*e*_ (mg/g)	*K*_*d*_ (mL/g)	*α*_*Li*_ */*_*Me*_
Li^+^	50	43.88	1.53	34.86	—
Na^+^	250	249.36	0.16	0.64	54.34
K^+^	250	249.24	0.19	0.76	45.74
Ca^2+^	250	248.92	0.27	1.10	31.56
Mg^2+^	250	248.76	0.31	1.25	27.98

### 3.5 Effect of initial pH

**FIGURE 8:**
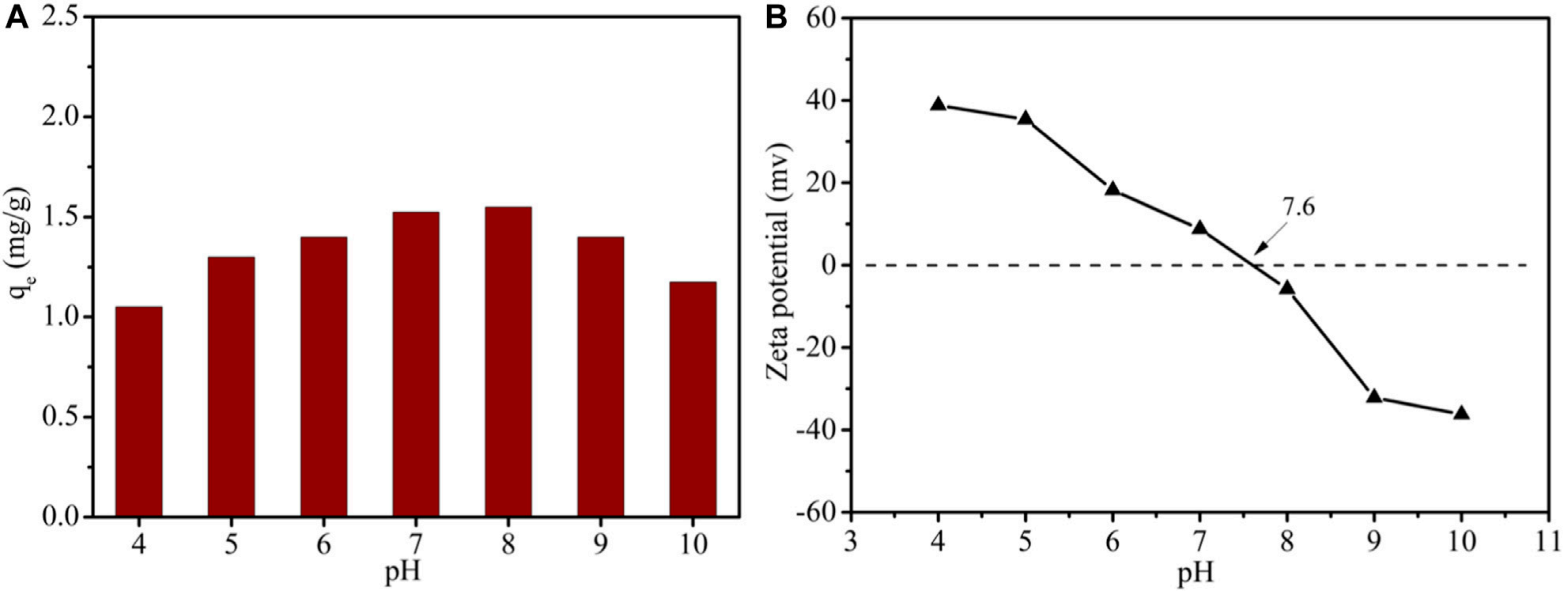
Li^+^ adsorption in different pH by LDH-Si-BX **(A)**; Zeta potentials of LDH-Si-BX **(B)**.

To investigate the pH sensitivity of LDH-Si-BX in the adsorption of Li^+^, a series of 50 mg/L Li^+^ solutions, which were adjusted to pH levels of 4.0–10.0, were used to test adsorption, as shown in Figure 8A. It was clear to see that the Li^+^ adsorbed by LDH-Si-BX had significant pH sensitivity. The higher adsorption capacity of 1.55 mg/g and 1.50 mg/g were observed at pH = 8 and pH = 7, which was close to the point of zero charges (PZC) of 7.6. When the pH value decreased from 7 to 4, the adsorption amount showed a significantly decreasing trend and dropped to 1.05 mg/g. Moreover, it could be noted that the adsorption decreased to 1.15 mg/g when the pH was increased from 8 to 10. This pH-dependent behavior indicated that the neutral medium was favorable for Li^+^ adsorption by LDH-Si-BX.

The zeta potentials of LDH-Si-BX are shown in Figure 8B. It was found that the isoelectric point (IEP) of LDH-Si-BX was determined as 7.6. The surface of LDH-Si-BX was positively charged by being protonated when the pH < pH _IEP_, which was not favorable for lithium-ion adsorption due to charge repulsion. When the pH > pH_IEP_, the surface of the adsorbents showed a negative charge, however, the adsorption performance of LDH-Si-BX for Li^+^ still presented a decreasing tendency, which might be attributed to the LiCl·2Al(OH)_3_·nH_2_O of LDH-Si-BX being converted to the Al(OH)_3_ (Zhong et al., 2020). It could be recognized that a neutral environment was conducive to the adsorption of Li^+^.

### 3.6 Adsorption and recycling in the real brines

**FIGURE 9:**
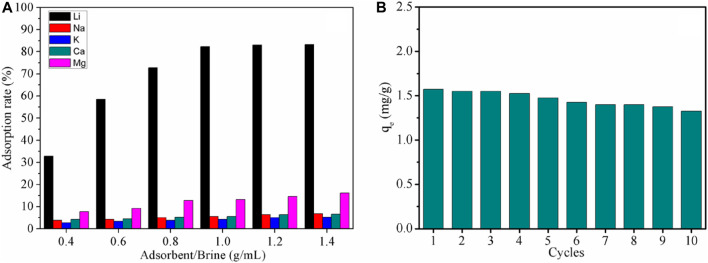
Adsorption properties under different solid-liquid ratios **(A)** and 10 cycles regeneration performance of LDH-Si-BX **(B)**.

To further investigate the adsorption performance of LDH-Si-BX on lithium ions in real brines, the study applied LDH-Si-BX to the adsorption of oilfield water. The adsorption kinetics are shown in Supplementary Figure S1 of the Supplementary Material, and the results show that the adsorption reached equilibrium within 180–200 min and was in accordance with the proposed second-order kinetic model (Supplementary Table S3 of the Supplementary Material). The data from the adsorbent/brines ratio study (Figure 9A) showed that the adsorption of lithium ions in oilfield brines by LDH-Si-BX reached 83.3% when the solid-liquid ratio was 1.0 g/mL. In addition, by comparing the adsorption rates for Li^+^, Na^+^, K^+^, Mg^2+^, and Ca^2+^ of LDH-Si-BX, it could be found that the adsorption capacity of LDH-Si-BX for Li^+^ was significantly stronger than that of the other four ions, indicating that LDH-Si-BX also possesses Li^+^ selectivity in the actual brines.

Figure 9B illustrates the changes in the adsorption amount of Li^+^ by LDH-Si-BX after 10 cycles of adsorption-desorption, and the results showed that a decrease in adsorption capacity occurred during the regeneration of the adsorbent, which could be attributed to the adsorption sites being occupied by Mg^2+^ in the brine (Zhong et al., 2020; Zhong et al., 2021). The adsorption amount of Li^+^ by LDH-Si-BX decreased by 16.8% after 10 regenerations, and it was observed that LDH-Si-BX possessed a certain regeneration capability.

### 3.7 Mechanism analysis

To further understand the Li^+^ adsorption mechanism by LDH-Si-BX, x-ray photoelectron spectroscopy (XPS) was used to characterize and analyze the compositional and structural changes of the adsorbents before and after Li^+^ adsorption (LDH-Si-BX-Li).

**FIGURE 10:**
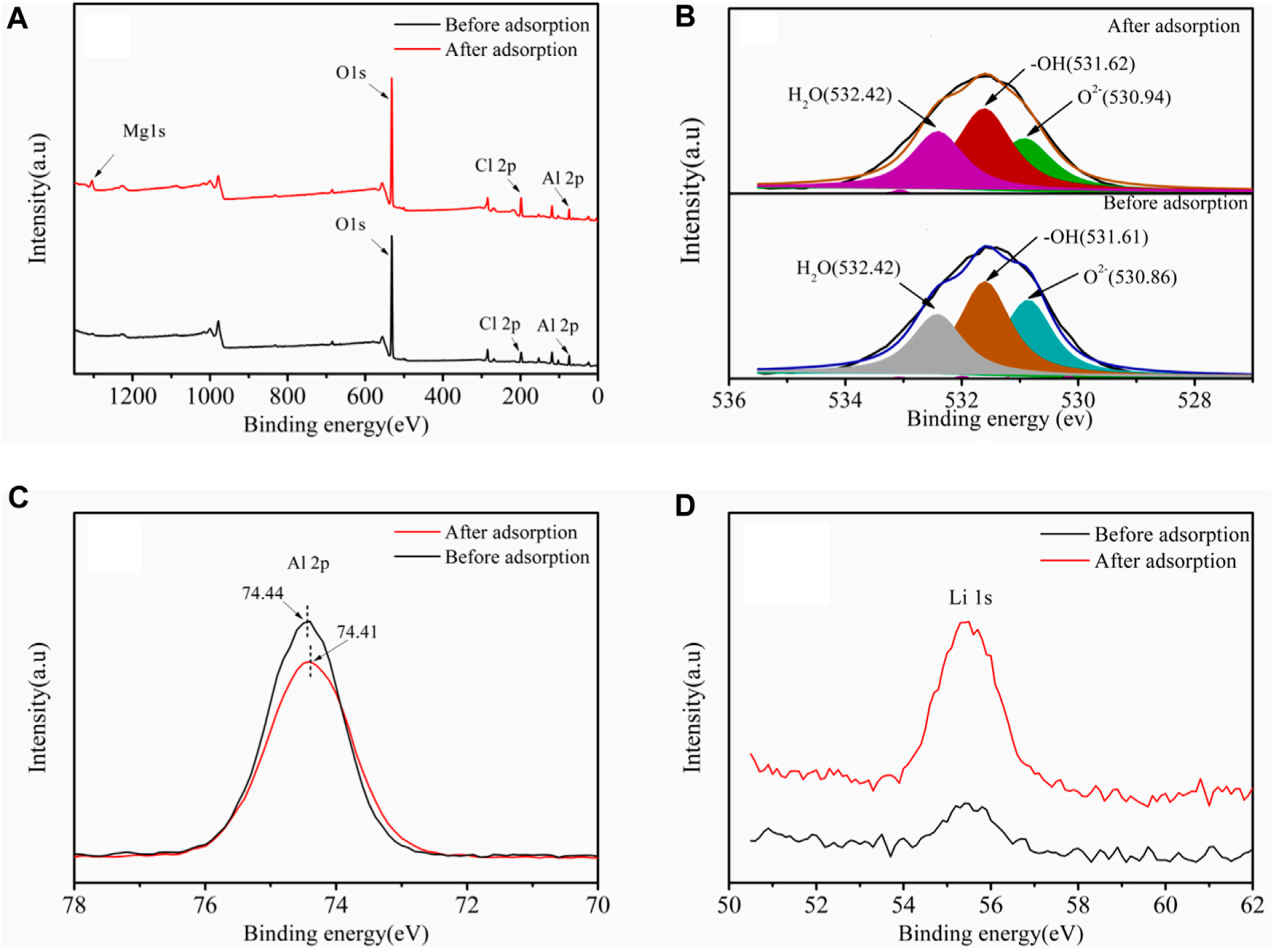
XPS spectra of LDH-Si-BX and LDH-Si-BX-Li: Survey spectra **(A)**, O 1s spectrum **(B)**, Al 2p spectrum **(C)**, Li 1s spectrum **(D)**.

As seen in Figure 10B, the three peaks of O^2-^, -OH, and H_2_O (Sleiman et al., 2016) could be combined to fit the high-resolution spectra of O1s for LDH-Si-BX and LDH-Si-BX-Li, and the fitting parameters results (Table 5) demonstrate that the binding energy of -OH, and H_2_O did not change significantly before and after adsorption, while the binding energy of O^2-^ increased from 530.86 eV to 530.94 eV after adsorption. In addition, as can be seen in Figure 10C, the binding energy of Al decreased from 74.44eV to 74.41eV. This phenomenon indicated that the influence of -OH and H_2_O in the LDH-Si-BX structure was not significant in the process of Li^+^ adsorption, while the O in the oxide structure played a vital role. Since the LiCl•2Al(OH)_3_•nH_2_O of LDH-Si-BX was the main substance for Li^+^ adsorption, it could be inferred that the O in the structure of Al-O played an important part in binding with Li^+^.

**TABLE 5:** Fitting parameters of O 1s peak of LDH-Si-BX and LDH-Si-BX-Li.

Samples	Chemical states	Binding energy (eV)
LDH-Si-BX	O^2-^	530.86
	-OH	531.61
	H_2_O	532.42
LDH-Si-BX-Li	O^2-^	530.94
	-OH	531.62
	H_2_O	532.42

Moreover, compared to the Li 1s high-resolution scan spectra (Figure 10D) of LDH-Si-BX and LDH-Si-BX-Li, the peak intensity of LDH-Si-BX-Li was significantly greater than that of LDH-Si-BX, which improved the adsorption of Li^+^ by LDH-Si-BX.

## 4 Conclusion

The prepared LDH-Si-BX showed that it had a certain Li + adsorption capacity and exhibited a favorable regeneration and recycling ability in the batch experiments. Adsorption studies showed that the Li+adsorption kinetics followed the pseudo-second-order model. The Langmuir model could describe well the adsorption data at different concentrations with a saturated Li^+^ adsorption capacity of 1.70 mg/g at 298K. In the presence of Na^+^, K^+^, Ca^2+^, and Mg^2+^, LDH-Si-BX also exhibited favorable selective adsorption for Li^+^. The pH study revealed that favorable Li^+^ adsorption could be obtained in a neutral environment. The adsorption and regeneration experiments manifested that the adsorption capacity of the adsorbent for Li^+^ decreased by 16.8% after 10 regenerations, which also indicated the Li^+^ adsorption potential by LDH-Si-BX in real brines. Therefore, it can be concluded that is suitable to recover Li^+^ from brines.

## Data Availability

The raw data supporting the conclusion of this article will be made available by the authors, without undue reservation.
