# Supplementary material for: Adsorption performance and mechanism of Li+ from brines using lithium/aluminum layered double hydroxides-SiO2 bauxite composite adsorbents
Source: Front Chem. 2023 Oct 25;11:1265290. doi: 10.3389/fchem.2023.1265290 (PMC10634247; doi:10.3389/fchem.2023.1265290)
Supplement: Supplementary file 1 [file DataSheet1.docx]

Supplementary material


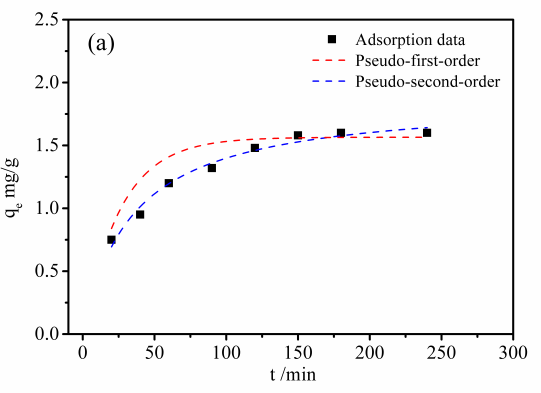


**Figure S1** Adsorption kinetic of Li^+^ onto LDH-Si-BX for real brine

Table S1 Chemical composition of raw bauxite in this study..

| Components | Al_2_O_3_ | SiO_2_ | Fe_2_O_3_ | TiO_2_ | Na_2_O | K_2_O | MgO | CaO | P_2_O_5_ | LOI |
| --- | --- | --- | --- | --- | --- | --- | --- | --- | --- | --- |
| **Wt%** | 48.82 | 34.26 | 2.26 | 1.06 | 0.12 | 0.24 | 0.84 | 0.18 | 0.12 | 10.62% |

**Table S2** Thermodynamic parameters for the Li+ adsorption on LDH-Si-BX

| Temperature (K) | lnK | Δ*G^o^*（kJ/mol） | Δ*H^o^(*kJ/mol) | Δ*S^o^*(J/(mol·K)) |
| --- | --- | --- | --- | --- |
| 298 | 7.195 | -17.826 |  |  |
| 308 | 7.349 | -18.208 | 9.602 | 92.102 |
| 318 | 7.426 | -18.399 |  |  |

**Table S3** Kinetic parameters for Li^+^ adsorption on LDH-Si-BX for real brine

| Concentration (mg/L) | Pseudo-first-order model | | Pseudo-second-order model | | | | Actual adsorption capacity (mg/g) |
| --- | --- | --- | --- | --- | --- | --- | --- |
|  | k_1_ (min^-1^) | R2 |  | k_2_ | q_e_ | R2 |  |
| 60.2 | 0.038 | 0.922 |  | 0.016 | 1.58 | 0.986 | 1.52 |
